# Hypercapnic cerebral edema presenting in a woman with asthma: a case report

**DOI:** 10.1186/1752-1947-5-192

**Published:** 2011-05-20

**Authors:** Ryan R Joyce, William T McGee

**Affiliations:** 1 Baystate Medical Center, The Western Campus of the Tufts University Medical School, 759 Chestnut Street, Springfield, MA 01199, USA

## Abstract

**Introduction:**

Common causes of non-traumatic acute cerebral edema include malignant hypertension, hyponatremia, anoxia, and cerebral vascular accident. The computed tomographic images and data obtained during care of the patient described in this case report provide evidence that hypercarbia can cause increased intracranial pressure and coma without permanent brain injury. Partial pressure of carbon dioxide evaluation for coma is essential to provide faster diagnosis and therapeutic correction in certain common critical disease states. We present the case of a patient in a coma associated with cerebral edema during a typical asthma exacerbation with hypercapnic respiratory failure.

**Case presentation:**

An obese 63-year-old African American woman with asthma presented to our hospital with facial swelling and shortness of breath. Immediately following intubation for hypercapnic respiratory failure, she was noted to have a dilated, unresponsive right pupil. An emergent computed tomographic head scan revealed that she had increased intracranial pressure. A neurosurgeon agreed with the computed tomography interpretation and recommended no surgical intervention. The patient's respiratory acidosis was corrected with ventilatory management over several hours in the intensive care unit. Nine and one-half hours later a follow-up head computed tomographic scan was read as normal without cerebral edema. At 12 hours, the patient's right pupil was 5 mm in diameter and reactive. By 24 hours, her pupils were symmetrically equal and reactive. Her symptoms had improved, and she was extubated. A brain magnetic resonance imaging scan revealed no abnormalities.

**Conclusion:**

Alteration of consciousness related to hypercapnia during respiratory failure is not generally thought to be related to cerebral edema. Respiratory acidosis resulting from hypercarbia is known to produce carbon dioxide narcosis and coma, but no current treatment algorithm suggests that rapid hypercapnia correction can be critical to neurologic outcome. To the best of our knowledge, our case is a unique example of the physiological changes that may occur in relation to arterial carbon dioxide concentration in the normal brain in the setting of typical hypercapnic respiratory failure. Correction of respiratory acidosis reversed the neurologic symptoms and physiology causing cerebral edema and coma in our patient. Rare similar cases have been sporadically reported in the medical literature, typically in children. Our case is also unusual in that rapid deterioration and clinical status were directly observed on simultaneous computed tomographic scans. Had this patient been found unresponsive, or had she had brief respiratory or cardiac arrest, the scan could have been interpreted as global anoxic injury leading to a different therapeutic course.

## Introduction

The correlation between physical examination, laboratory data, and radiographic imaging before and after hypercapnia therapy for respiratory failure provides documentation of changes in partial pressure of carbon dioxide (PaCO_2_) and physiology of the brain capable of producing coma and its reversal. Prior cases of this phenomenon have been sporadically reported in the medical literature, typically in children with acute, severe respiratory acidosis without brain imaging or arterial blood gas (ABG) data [1,2]. Cerebral edema is not generally cited as a cause of stupor and coma during typical hypercapnic respiratory failure in an uninjured brain. Our patient's rapid deterioration and clinical status were directly observed, and the computed tomography (CT) findings did not suggest any alternative diagnosis.

## Case presentation

**Figure 1:**
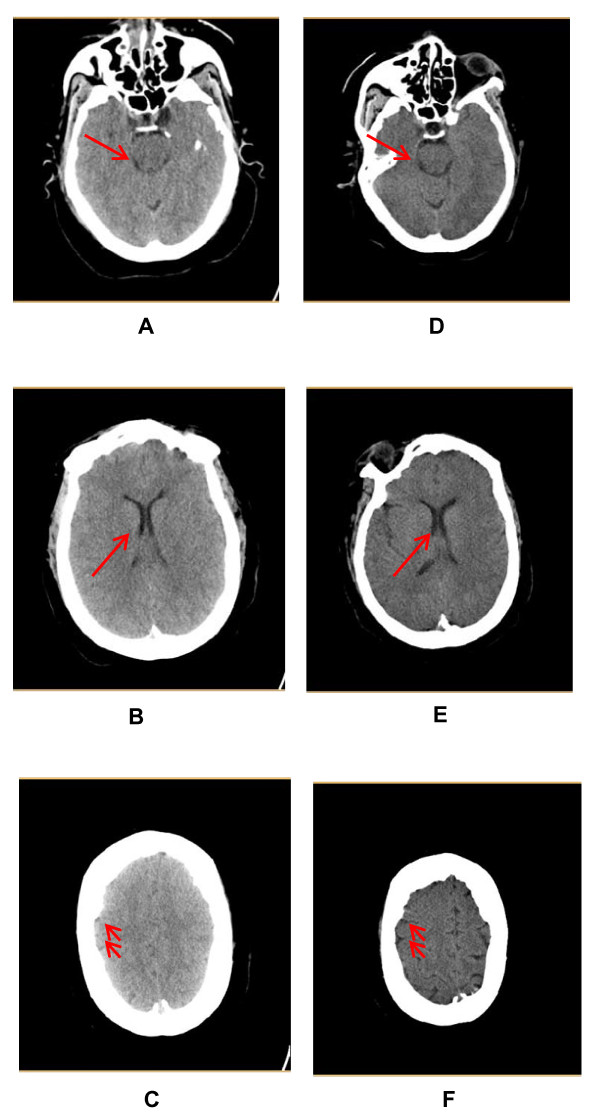
**(A-C) Pre-CO_2 _correction**. **(A) **Basal cisterns. **(B) **Ventricles. **(C) **Sulci. **(D through F) **Post-CO_2 _correction. **(D) **Basal cisterns. **(E) **Ventricles. **(F) **Sulci.

An obese 63-year-old African American woman presented to our hospital with sudden onset of bilateral facial and tongue swelling associated with progressive shortness of breath. Her medical history included asthma, hypertension, congestive heart failure, obstructive sleep apnea, and obesity. She has minor allergies to certain fruits but no prior history of anaphylaxis. Her initial vital signs were blood pressure 127/67 mmHg, pulse rate 98 beats/minute, and 100% oxygen saturation on 6L nasal cannula with a respiratory rate of 18 breaths/minute. Immediate therapy in the emergency department included epinephrine, diphenhydramine, and solumedrol for presumed anaphylaxis; intubation; and mechanical ventilation. Extensive laryngeal edema as well as a dilated, unresponsive right pupil were pertinent positive findings on physical examination. An emergent CT scan of her head showed bilateral effaced sulci with both small ventricles and basilar cisterns. These findings were consistent with increased intracranial pressure (ICP) (Figures 1A through 1C). The patient was admitted to the intensive care unit (ICU).

Initial ABG analysis revealed severe respiratory acidosis, which was corrected over the course of several hours (Table 1). During her initial hours in the ICU, she experienced persistent respiratory acidosis despite being on controlled volume ventilation at a rate of 12 beats/minute. Serial ABG analyses were monitored, and her respiratory rate was adjusted as shown in Table 1. A neurosurgeon was consulted, agreed with the CT interpretation, and recommended no surgical therapy. She was continued on scheduled methylprednisolone, diphenhydramine, and albuterol in the ICU as well. Mannitol was not administered, as she had no history or laboratory indications suggesting that an osmotic imbalance was causing her edema. Her physical examination continued to show a dilated, unresponsive right pupil with a pinpoint, yet reactive, left pupil. Disc margins were blurred bilaterally without hemorrhages or exudates. She remained unresponsive.

**Table 1:** Serial data regarding ABGs, ventilator settings, and changes made after ABGs were obtained for the duration of mechanical ventilation, including the initial pre-intubation ABG^a^

		Time				
	
	Day 1					Day 2
	
	03:00	06:43	08:52	10:56	12:54	16:34
pH	7.22	7.23	7.41	7.70	7.64	7.42
pCO_2_	85	89	59	29	36	52
pO_2_	109	82	67	84	75	115
HCO_3_^-^	33	40	40	36	39	35
RR	12	12	18	21	16	20
*V*_t_	600	600	500	500	Varied	Varied
F_i_O_2_	50	50	50	50	40	40
Change made after ABG	Pre-intubation initial CT scan	RR increased to 22 breaths/minute	No change	Spontaneous breathing observed	Follow-up CT scan performed	Extubation

^a^ABG, arterial blood gas; pCO_2_, partial pressure of carbon dioxide; pO_2_, partial pressure of oxygen; HCO_3_^-^, bicarbonate; RR, respiratory rate; *V*_t_,; F_i_O_2_, fraction of inspired oxygen; CT, computed tomography.

Eight hours after intubation, the patient's physical and neurological findings were unchanged. Nine and one-half hours later the patient's right pupil was noted to be 5 mm in diameter and was now reactive. The left pupil was unchanged. Follow-up head CT performed at this time (Figures 1D through 1F) was normal without cerebral edema. After 24 hours her pupils had become symmetrically equal and reactive. The remainder of her neurological examination remained normal. She awoke and was extubated. MRI of the brain revealed no pathology.

Two weeks following her discharge from the hospital, she was seen in follow-up as an outpatient in our neurosurgery department. She denied having headaches or double vision. Her cranial nerve examination, deep tendon reflexes, and bilateral motor sensory function were normal, as was the assessment of her cognitive function.

## Discussion

Alteration of consciousness related to hypercapnia during respiratory failure is not generally thought to be related to cerebral edema. The severe respiratory acidosis that results from hypercarbia is known to produce CO_2 _narcosis with coma [2]. No current treatment algorithm suggests that treatment of hypercapnia in an obtunded patient with hypercapnic respiratory failure is critical to neurologic outcome. In fact, permissive hypercapnia has been recommended to address ventilator-induced lung injury in some cases of respiratory failure [3,4].

The differential diagnosis for a single dilated pupil includes eye or sympathetic trunk trauma, ocular anti-cholinergic or α-adrenergic agents, and cranial nerve III injury or compression from intracranial hypertension [5]. In our patient, there was no trauma or use of mydriatic agents. Considering the changes noted on the first CT scan (Figures 1A through 1C), the most likely explanation for unilateral mydriasis was increased ICP causing uncal herniation and unilateral cranial nerve III compression [5]. The elevated concentration of carbonic acid and hydrogen ions from dissolved CO_2 _produces dilatation of the cerebral arteries, leading to increased cerebral blood flow and elevation of ICP [2,6]. The direct correlation between paCO_2 _causing cerebral vascular dilation has been well-documented in animal models also producing increased ICP [6].

Brain edema is caused by an increase in intracellular or extracellular water. Damage from ischemia, anoxia, and cytotoxins causes injury to neuronal and vascular cells, leading to cellular swelling [7]. Hypoxia is known to be a direct cause of cerebral edema [8]; however, our patient's initial partial pressure of oxygen saturation suggests that this was not a factor in her condition. Forced exhalation may increase ICP by limiting cerebral venous drainage. Although not specifically observed or commented upon prior to intubation in our patient, this has been reported as a cause of subarachnoid hemorrhage (SAH) in patients with severe asthma [9]. Once intubated, our patient was deeply sedated, and imaging did not reveal SAH. Hypercapnia and the resultant intracellular acidosis can produce a central nervous system depressive effect by itself [7]. In our patient, however, only elevated ICP explains the dilated pupil and cerebral edema. In this case, cerebral edema and its reversal were directly correlated with PaCO_2_. No other explanation is likely.

## Conclusion

Our patient's scenario provides a unique example of the physiological and anatomical changes that may occur in relation to arterial CO_2 _concentration in the normal brain. Treatment was targeted at correction of respiratory acidosis using ventilation management, which reversed the physiology that caused her cerebral injury and led to a good outcome. Over the course of nine and one-half hours, CO_2 _correction was documented by both clinical and radiologic improvement in cerebral edema. Cerebral edema should be considered as a cause of obtundation complicating respiratory failure that can respond to CO_2 _correction.

## Abbreviations

CT: computed tomography; ICP: intracranial pressure; ICU: intensive care unit.

## Consent

Written informed consent was obtained from the patient for publication of this case report and any accompanying images. A copy of the written consent is available for review by the Editor-in-Chief of this journal.

## Competing interests

The authors declare that they have no competing interests.

## Authors' contributions

RRJ and WTM analyzed and interpreted the patient data regarding hypercapnia, anaphylaxis, and increased intracranial pressure. Both authors were major contributors to writing this manuscript. Both authors read and approved the final manuscript.
